# Chromatic Aberration Correction in Harmonic Diffractive Lenses Based on Compressed Sensing Encoding Imaging

**DOI:** 10.3390/s24082471

**Published:** 2024-04-12

**Authors:** Jianying Chan, Xijun Zhao, Shuo Zhong, Tao Zhang, Bin Fan

**Affiliations:** 1 Thin Film Optical Camera General Room, Institute of Optics and Electronics, Chinese Academy of Sciences, Chengdu 610051, China; chenjianying22@mails.ucas.ac.cn (J.C.);; 2 The Center of Advanced Optical Manufacturing, Institute of Optics and Electronics, Chinese Academy of Sciences, Chengdu 610209, China

**Keywords:** compressive sensing, diffractive achromatic, computational imaging

## Abstract

Large-aperture, lightweight, and high-resolution imaging are hallmarks of major optical systems. To eliminate aberrations, traditional systems are often bulky and complex, whereas the small volume and light weight of diffractive lenses position them as potential substitutes. However, their inherent diffraction mechanism leads to severe dispersion, which limits their application in wide spectral bands. Addressing the dispersion issue in diffractive lenses, we propose a chromatic aberration correction algorithm based on compressed sensing. Utilizing the diffractive lens’s focusing ability at the reference wavelength and its degradation performance at other wavelengths, we employ compressed sensing to reconstruct images from incomplete image information. In this work, we design a harmonic diffractive lens with a diffractive order of M=150, an aperture of 40 mm, a focal length $f_{0}=320$ mm, a reference wavelength $\lambda_{0}=550$ nm, a wavelength range of 500–800 nm, and 7 annular zones. Through algorithmic recovery, we achieve clear imaging in the visible spectrum, with a peak signal-to-noise ratio (PSNR) of 22.85 dB, a correlation coefficient of 0.9596, and a root mean square error (RMSE) of 0.02, verifying the algorithm’s effectiveness.

## 1. Introduction

Traditional optical systems, designed to eliminate aberrations with multiple lenses, are complex, bulky, and expensive, failing to meet weight requirements [1]. Diffractive lenses, with their micrometer-scale thickness, offer advantages of ultra-thinness and light weight. A single diffractive lens can intricately control the light field, holding the potential to replace traditional refractive and reflective systems. However, their inherent diffraction mechanism leads to significant chromatic dispersion, limiting high-precision wide-spectrum imaging applications [2]. Traditional solutions involve adding a reverse power diffractive lens to correct chromatic aberrations or designing multi-layered diffractive lens structures to enhance efficiency [3]. In addition to improvements to element structures, chromatic aberration correction can be achieved through image processing algorithms, which is a central concept in computational imaging [4]. In computational imaging systems, the optical system can be incomplete, and high-quality images can be recovered from system-captured data through image reconstruction algorithms. In recent decades, computational imaging technology has been applied in various fields, such as single-pixel imaging [5,6,7], structured light 3D imaging [8], lensless imaging [9,10,11], coded imaging [12,13], and hyperspectral imaging [14,15,16,17], becoming a research hotspot in the field of optical imaging. Introducing computational imaging technology into diffractive lens systems significantly enhances optical system design freedom and simplifies system structures. Nikonorov et al. proposed a three-channel chromatic aberration correction algorithm, blurring and sharpening deblurred images in other channels for color correction and reconstructing Fresnel lens imaging results, but the recovered images still exhibited significant noise [18]. Peng et al. used a particle swarm algorithm to optimize the diffractive lens height map, reconstructing images based on cross-channel image priors, but the low diffraction efficiency resulted in foggy images [16]. Sitzmann et al. first introduced the framework for joint design of optical algorithms, obtaining the imaging data of optical systems through simulation [19]. This approach combines deep learning with backend image restoration algorithms, colloquially known as the end-to-end design framework. This framework has pioneered a new paradigm in computational imaging design. Since this introduction, there has been an abundance of related work, including multispectral imaging [20,21], depth estimation [22,23], and large field imaging [24]. Although the end-to-end design framework has achieved breakthroughs in optical device performance compared to traditional design methods, it still faces several challenges. These include dependency on datasets, the need for high computational power, and the inability to design large-aperture diffractive optical elements (to our knowledge, there are no diffractive lenses with an aperture larger than 2 cm currently available). Therefore, our work continues to employ the traditional approach of separating the design of optical components from backend algorithms. At the same time, the end-to-end design framework is limited to the high-cost photolithography process for fabricating diffractive optical elements. In our work, we opt for a cost-effective and straightforward approach by employing turning machining for the processing of optical components. Traditional image restoration algorithms include point spread function-based deblurring algorithms like Lucy–Richardson [25], cross-channel non-blind deconvolution [26], and cross-channel non-blind convex optimization deconvolution based on estimated point spread functions [18]. Diffractive lenses have undergone substantial evolution, transitioning from simple diffractive optical elements to sophisticated harmonic diffractive lenses. Harmonic diffractive lenses offer significant advantages over traditional diffractive lenses, including improved chromatic aberration control, higher diffraction efficiency, broader bandwidth operation, better system integration, increased manufacturing flexibility, and enhanced customization capabilities. Harmonic optical elements have interesting and useful optical properties, and they may be used for lightweight optical components in future space telescopes [27], remote sensing [28], and other applications [29].

**Figure 1:**
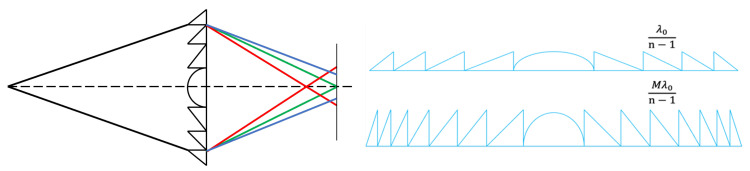
**Left** shows a schematic of chromatic dispersion in diffractive lenses; **right** illustrates the structure of a harmonic diffractive lens.

**Figure 2:**
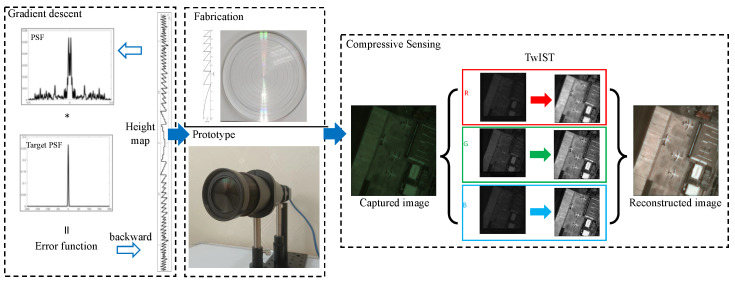
Overall workflow diagram of the work. We designed a 150th-order harmonic diffractive lens based on the gradient descent method. Given a randomly generated initial structure of a diffractive lens, we obtained the PSF of the current diffractive lens structure. Our goal was to make the PSF converge as closely as possible to a single point. We calculated the error between the current PSF and the target PSF, adjusting the structure of the diffractive lens using the gradient descent algorithm. The final optimized lens was then fabricated, a prototype system was assembled, and the image was captured. Utilizing the proposed image recovery algorithm based on compressed sensing, we achieved the effect of chromatic aberration correction.

In this work, we consider chromatic aberration as a result of different focal points across various spectral bands. For simplicity of discussion, we present a schematic diagram showing light of three different wavelengths (RGB) converging at different focal points after passing through the same diffractive lens as show in Figure 1. By performing full-focus restoration on individual bands and then merging them, we obtain an image with chromatic aberration correction and propose a novel image restoration method based on compressed sensing for chromatic aberration correction. The overall workflow is shown in Figure 2: First, we design a 150th-order harmonic diffractive lens based on gradient descent [28], conduct fabrication experiments, capture images, perform reconstruction based on three channels, focus the designed wavelength channel image, utilize compressed sensing for reconstruction in other incomplete channels, and successfully correct chromatic aberrations through simple iteration. We conducted both infield and outfield experiments; infield experiments involved displaying true images on a monitor, capturing these with a prototype system, and reconstructing the images to facilitate quantitative evaluation of the restoration results, such as PSNR. Outfield experiments entailed direct capture of natural landscapes for reconstruction, with the quality of restoration assessed solely through visual inspection by human observers.

## 2. Diffractive Lens Imaging Model

Diffractive lenses, as an integral component of modern optical engineering, exhibit two distinctive characteristics that set them apart from their refractive counterparts, namely multi-level diffraction and wavelength sensitivity, often referred to as dispersion, as illustrated in Figure 1 left. These features are not merely incidental but are fundamental to the operational principles and applications of diffractive optics. Multi-level diffraction, a hallmark of diffractive lenses, arises from their unique physical structure. Unlike traditional lenses, which rely on the continuous curvature of their surfaces to bend light, diffractive lenses achieve focus through the constructive and destructive interference of light waves. This is facilitated by the lens’s surface, which is etched or molded into multiple discrete levels or steps. Each level corresponds to a specific phase shift, orchestrating the light waves to converge at the focal point. This multi-level approach allows diffractive lenses to precisely control the phase of incoming light, enabling them to focus light efficiently and with a high degree of flexibility in design. As such, diffractive lenses can be engineered to achieve specific optical functions that would be challenging or impossible to accomplish with conventional refractive lenses. Wavelength sensitivity, or dispersion, is another critical aspect of diffractive lenses. This characteristic stems from the way diffractive lenses manipulate light, which is inherently dependent on the wavelength of the incident light. The diffraction efficiency of a lens—its ability to direct light towards the desired focal point—varies with the wavelength, leading to a phenomenon where different wavelengths are focused at slightly different positions. This dispersion effect can be a double-edged sword. On the one hand, it allows for the design of lenses that can selectively focus or filter light based on wavelength, which is advantageous for applications such as chromatic correction and spectral imaging. On the other hand, it necessitates careful design to mitigate unwanted chromatic aberrations in applications where uniform focus across a broad spectrum of wavelengths is desired. The interplay between multi-level diffraction and wavelength sensitivity defines the operational envelope and design considerations for diffractive lenses. These characteristics enable the lenses to be highly compact and lightweight, offer unique dispersion properties, and achieve high focusing efficiencies. However, they also pose challenges, particularly in terms of managing dispersion and designing for broadband applications. Advances in computational design and fabrication technologies continue to push the boundaries of what is possible with diffractive optics, enabling increasingly sophisticated optical devices that leverage the unique advantages of multi-level diffraction and wavelength sensitivity. In our work, we primarily address the dispersion issue with a post-process algorithm. According to scalar diffraction theory [30], the point spread function (PSF) can be expressed as:

(1) $$p\left(x,y;\lambda \right)=\frac{A}{\lambda z_{i}}\;\int \int \;P\left(u,v;\lambda \right)e^{-j\frac{2\pi }{\lambda z_{i}}\left(ux+vy\right)}\;du\;dv$$

where *A* is the amplitude constant, $z_{i}$ is the distance from the lens to the imaging plane, (u,v) are the coordinates on the lens plane, and P(u,v;λ) is the pupil function. The pupil function for a diffractive lens is:

(2) $$P\left(u,v;\lambda \right)=\mathrm{Circ}\left(u,v\right)e^{j\Phi (u,v)}$$

Circ(·) is the circular aperture function, and Φ(u,v) is the phase delay introduced at each point after passing through the lens. In our work, we acquire the desired imaging effects and PSF by optimizing the Φ(u,v) term. In Fourier optics, the incoherent imaging model is viewed as the convolution process of the optical system input i(x,y;λ) with the PSF:

(3) $$A\left(i\left(x,y;\lambda \right)\right)=i\left(x,y;\lambda \right)⊗{\left|p\left(x,y;\lambda \right)\right|}^{2}$$

Here, ⊗ denotes the two-dimensional convolution operation, and A(i(x,y;λ)) represents the chromatic aberration-affected image. For traditional diffractive lenses, the PSF p(x,y;λ) heavily depends on the wavelength, leading to significant dispersion. This wavelength dependence causes different focal lengths *f* for different wavelengths λ. For a diffractive lens designed for wavelength $\lambda_{0}$ and focal length $f_{0}$, the focal length for other wavelengths satisfies:

(4) $$\lambda f=\lambda_{0}f_{0}$$

Ideal imaging channels the camera capture through image sensors. Image sensors vary in sensitivity to different wavelengths, requiring the convolution-derived image to be multiplied by a spectral response function, which is an intrinsic characteristic of the sensor. Generally, cameras capture RGB images (the central wavelengths of RGB are 640 nm, 550 nm, and 460 nm, respectively, with the total coverage range including the wavelength band of our designed diffractive lens, which is 500–800 nm), and our post-process algorithm is also based on RGB images. Therefore, in designing the diffractive lens, the wavelength range covers each color channel in RGB. In this case, the continuous PSF can be discretized into three channels. The entire forward imaging model can be written as:

(5) $$b_{c}=p_{c}\left(x,y\right)⊗p_{c}\left(x,y\right),\;c\in \left\{r,g,b\right\}$$

In our work, we designed a diffractive lens with a reference wavelength for the G channel. For the R and B channels, severe image degradation occurs, resulting in green-tinted captured images. The image captured on the reference focal plane is decomposed into R, G, B channels. From a single-channel perspective, dispersion can be understood as incomplete image information in the captured R and B channels, presenting a degraded effect. Retrieving the original image is a process of recovering complete information from limited data. Hence, we employ compressed sensing theory to recover individual channels and then superimpose the recovered channels to achieve chromatic aberration correction.

## 3. Harmonic Diffractive Lens Design

In the realm of scalar diffraction theory, diffractive lenses are traditionally modeled as phase masks, an approach that simplifies the interaction of light with the lens’s microstructured surface. This conventional model is predicated on the manipulation of the phase of light passing through the lens, employing a first-order diffractive surface to achieve the desired optical effects. In contrast, harmonic diffractive lenses represent a sophisticated advancement in this domain, characterized by their employment of a diffractive order *M* that exceeds unity. This higher-order approach allows for the modulation of light with greater finesse, resulting in a lens that can exhibit both diffractive and refractive properties. The structural distinction between harmonic diffractive lenses and their traditional counterparts is primarily attributed to the phase depth factor associated with the former. As *M* increases, the microstructure of the lens surface becomes more pronounced, reducing the number of annular zones required to achieve a specific optical effect. This relationship is illustrated in Figure 1 right, where the varying *M* values manifest in distinct lens profiles. Notably, at higher values of *M*, the harmonic diffractive lens increasingly resembles a traditional refractive lens in form, albeit with a complex microstructure that presents significant manufacturing challenges. Despite these challenges, the harmonic diffractive lens boasts a notable advantage in terms of diffraction efficiency. It is capable of achieving theoretical 100% efficiency at the design wavelength and at multiple harmonic wavelengths, surpassing the performance of traditional diffractive lenses, particularly in applications requiring wideband imaging. This efficiency is achieved through the strategic manipulation of the lens’s phase profile, as described by the phase compression formula:

(6) $$\phi_{doe}=\mathrm{mod}\left(\phi_{lens},2M\pi \right)$$

where $\phi_{lens}$ is the continuous phase of the refractive lens, and $\phi_{doe}$ is the compressed phase, with mod representing the modulo operation of $\phi_{lens}$ with 2Mπ. Based on the optical path difference formula, the corresponding sag height of the harmonic diffractive lens is:

(7) $$H_{doe}=\frac{\lambda_{0}\phi_{lens}}{(n_{\lambda }-1)2\pi }=\frac{M\lambda_{0}}{n_{\lambda }-1}$$

where $\lambda_{0}$ is the design wavelength.

In the advanced domain of optical engineering, diffractive lenses stand out for their ability to precisely manipulate light, offering innovative solutions for a wide range of applications from imaging systems to laser focusing devices. The fabrication of these lenses involves sophisticated techniques, predominantly photolithography and diamond turning, each with its own set of advantages and constraints. Photolithography, while precise, necessitates the discretization of the lens’s continuous height profile into multiple steps, thereby inflating the production costs and complexity. Conversely, diamond turning, a method we have employed in our work, leverages direct machining to achieve the desired surface profile, requiring detailed parameterization of the lens design for accurate fabrication. Our focus is on the development of a harmonic diffractive lens, distinguished by its diffractive order M, and on optimizing the harmonic diffractive lens’s continuous surface in order to enhance its optical performance. The key characteristics of this lens are its ability to achieve near-perfect diffraction efficiency at the design wavelength and its harmonics. The design process for such a lens necessitates a comprehensive understanding of its geometric and optical properties, starting with the determination of the maximum radius $R_{max}$ of the annular zones, which is essential for defining the lens’s aperture. The formula for $R_{max}$ is given by:

(8) $$R_{max}=\sqrt{N^{2}{\left(M\lambda_{0}\right)}^{2}+2Nf_{0}M\lambda_{0}}$$

where *N* represents the number of annular zones, $\lambda_{0}$ the design wavelength, $f_{0}$ the focal length, and *M* the diffractive order. This equation is pivotal for laying out the spatial arrangement of the annular zones, which are integral to the lens’s functionality. Following the spatial definition, the structural height parameter $H_{doe}$ for each annular zone is calculated. This parameter not only influences the focusing ability of the lens but also its efficiency in light manipulation across different wavelengths. The structural height parameter $H_{doe}$ is determined by the equation:

(9) $$H_{doe}=\frac{c_{i}R_{max}^{2}}{1+\sqrt{1-\left(1+k\right)c_{i}^{2}R_{max}^{2}}}+z_{off_{i}}$$

where $c_{i}$, $z_{off_{i}}$ represent the curvature and axial offset of the *i*th annular zone, respectively, and *k* is the conic coefficient.

**Figure 3:**
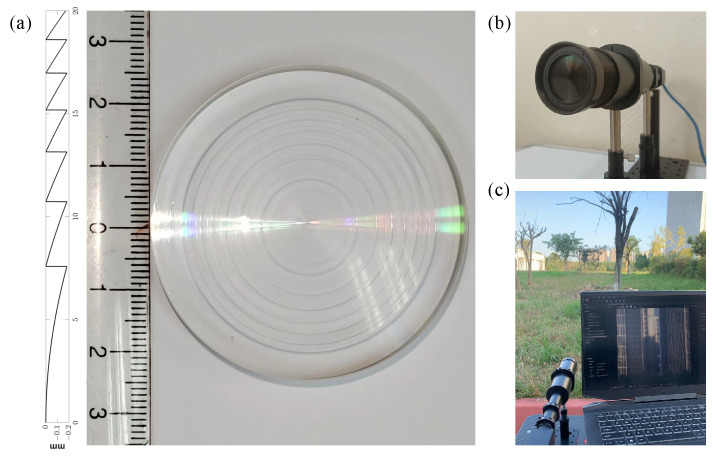
(**a**) Height map of the harmonic diffractive lens obtained through the gradient descent method, fabrication and prototype assembly of the device; (**b**) fabrication of the optimized height map and assembly of the prototype; (**c**) experimental setup for outdoor scene photography.

In our design endeavor, we aimed to fabricate a harmonic diffractive lens with a diffractive order of M=150 featuring an aperture of 40 mm, focal length $f_{0}=320$ mm, and design wavelength $\lambda_{0}=550$ nm and comprising N=7 annular zones. This design was meticulously optimized over 200 iterations using a gradient descent method, a process that underscored the intricacies involved in balancing the physical constraints with optical performance objectives. The outcome of this optimization was visually represented in the contour shown in Figure 3a, which encapsulates the nuanced surface profile necessary for achieving the lens’s design goals.

**Figure 4:**
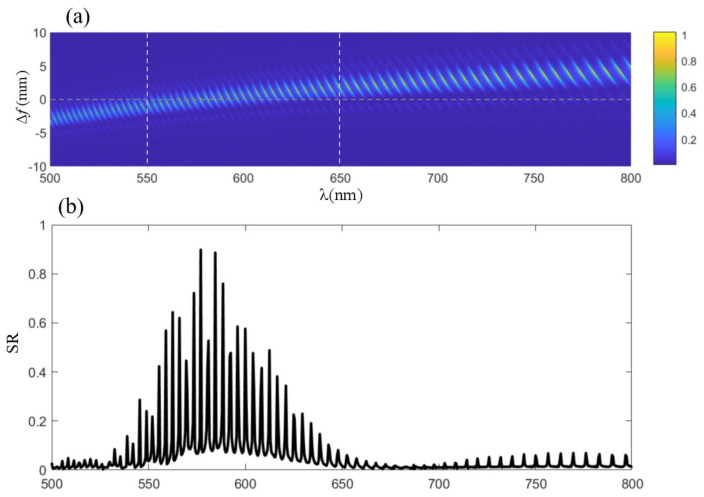
SR of our designed harmonic diffractive lens. The lens demonstrates superior focusing performance between 550 nm and 600 nm, exhibiting a performance peak as depicted in Figure (**a**), which correlates with the minimal defocus amount, f+Δf where Δf is zero, signifying optimal focusing at the design wavelength. As the wavelength deviates from this value, a shift in focal length is observed, accompanied by a reduction in diffraction efficiency, emphasizing the wavelength-dependent behavior of diffractive lenses. There is a good SR (greater than 0.8) in the range of 500—600 nm, as shown in (**b**).

To assess the lens’s focusing capabilities across a spectrum of wavelengths, the Strehl ratio (SR) was employed as a benchmark. The SR is a critical measure of optical performance, with a value of 80% denoting the diffraction limit, and values exceeding 95% indicating an almost aberration-free system. Our findings, depicted in Figure 4b, demonstrate that the lens exhibits superior focusing performance between 550 nm and 600 nm, in alignment with our design intentions. This performance peak, as illustrated in Figure 4a, correlates with the minimal defocus amount f+Δf, where Δf is zero, indicating optimal focusing at the design wavelength. As the wavelength diverges from this value, a shift in focal length is observed, accompanied by a reduction in diffraction efficiency, underscoring the wavelength-dependent behavior of diffractive lenses. The development of the harmonic diffractive lens, with its high diffractive order and optimized annular zone structure, represents a significant advance in optical engineering. The meticulous design and fabrication process, rooted in a deep understanding of optical physics and material science, illustrates the potential of such lenses in pushing the boundaries of what is achievable in light manipulation. This work not only contributes to the field by providing a novel lens design but also sets a precedent for future research in the pursuit of high-efficiency, wideband optical components. Through this endeavor, we have showcased the synergy between theoretical modeling, computational optimization, and precision manufacturing, highlighting the intricate balance required to translate complex optical concepts into tangible, high-performance optical devices.

## 4. Compressive Sensing

Compressed sensing (CS), a concept introduced by CANDÈS E and TAO T, leverages the sparsity of signals to reconstruct them from significantly fewer samples than required by the Nyquist sampling theorem. The core idea is that if a signal is sparse in a certain basis (i.e., most coefficients are zero or near zero), sufficient information can be captured through fewer non-adaptive linear measurements, allowing for accurate reconstruction of the original signal. A critical condition in this methodology is the restricted isometry property (RIP), expressed as:

(10) $$\left(1-\delta_{k}\right){\left||x|\right|}_{2}^{2}\leq {\left||Ax|\right|}_{2}^{2}\leq \left(1+\delta_{k}\right){\left||x|\right|}_{2}^{2}$$

where *k* represents the sparsity of the signal, *A* is the measurement matrix, $\left|\right|\cdot {\left|\right|}_{2}$ denotes the L2 norm, and $\delta_{k}$ is a positive number less than 1. Matrix *A* is said to satisfy the RIP condition if it fulfills this inequality for all *k*-sparse vectors *x*. However, verifying the RIP condition for a given measurement matrix in practical applications presents considerable challenges. Engineers and researchers often focus more on the pragmatic aspects, such as the volume of measurement data and the sparsity level of the signal under consideration. In the context of image recovery, two pivotal factors—sparsity and the incoherence of the measurement matrix—stand out as critical to the success of compressed sensing algorithms. In the specialized domain of imaging systems, the work of Wu et al. [11] showcased an innovative application of CS theory. They demonstrated that in a single diffractive lens imaging system, the system’s point spread function (PSF) can be effectively utilized as the measurement matrix. In a single diffractive lens system, the following imaging relationship formula exists:

(11) $$y=\frac{1}{2}F^{-1}H_{T}Fx$$

where *y* represents the system output image, *x* represents the source image, F and $F^{-1}$ respectively represent the Fourier transform and the inverse Fourier transform, and $H_{T}$ represents the transfer function. Further, we can express this as y=Kx, where $K=F^{*}\Sigma F$. For details, see [11].

This application underscores the versatility of CS theory in adapting to various practical scenarios where the measurement matrix may arise from the physical properties of the imaging system itself. The PSF, characterizing how a system responds to a point source or point object, inherently encodes information about the system’s resolution and imaging characteristics. By leveraging the PSF as the measurement matrix, the diffractive lens imaging system embodies the CS principles, enabling it to capture and reconstruct high-quality images from a number of measurements that defy conventional expectations. This approach not only highlights the adaptability of CS theory to diverse engineering challenges but also opens new avenues for enhancing imaging system performance through the strategic exploitation of signal sparsity and measurement incoherence. Our work builds upon this foundational research, applying it to the correction of chromatic aberration in single diffractive lens systems.

## 5. Algorithm Recovery Model

**Figure 5:**
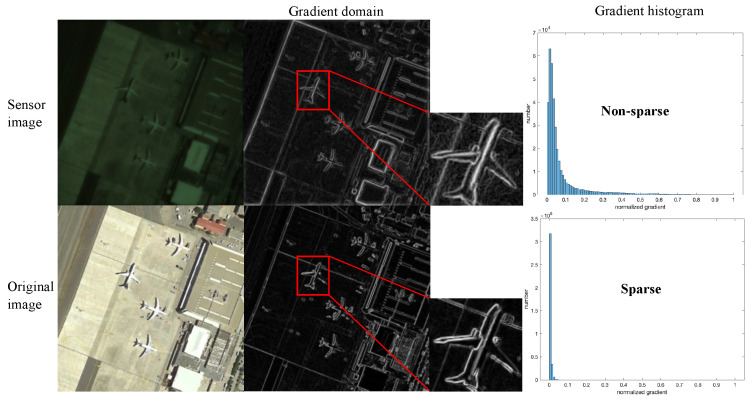
An analysis of chromatic and achromatic images in the gradient domain is conducted, where the achromatic image exhibits sparsity in the gradient domain, while the chromatic image shows non-sparsity. This meets the conditions of compressed sensing for image recovery.

Image recovery stands as a cornerstone in the domain of signal processing and computational imaging, addressing the challenge of reconstructing a high-quality image from degraded observations. This task is emblematic of linear inverse problems, a category characterized by the need to invert a known linear process that has been applied to the signal or image of interest. The quintessence of solving these problems lies in the formulation of an optimization problem, where the objective is to minimize a convex function that encapsulates both fidelity to the observed data and regularization terms to impose prior knowledge or assumptions about the solution. The optimization problem can be succinctly described by the following equation:

(12) $$f\left(x\right)=\arg\min\left(\frac{1}{2}||y-{Kx||}^{2}+\lambda \Phi \left(x\right)\right)$$

where *K* represents the linear operation mapping target data *x* to observed data *y*, ||·|| denotes the norm, λ∈[0,+∞[ is the regularization parameter, and Φ(·) represents the regularization method. The choice of regularization is pivotal, with common approaches including L1, L2, and total variation (TV) regularization, each suited to different aspects of image characteristics. L1 and L2 regularization focus on the magnitude of the image coefficients, promoting sparsity and smoothness, respectively. In contrast, TV regularization, especially pertinent to our work, excels in preserving edges while promoting smoothness within homogeneous regions of the image. This method proves particularly effective in the context of diffractive lens imaging, where chromatic dispersion introduces color bias and blurring, manifesting as sparsity in the gradient domain. TV regularization can be categorized into isotropic and anisotropic forms, mathematically represented as:

(13) $$\Phi_{iTV}\left(x\right)=\sum_{i}\sqrt{{\left(\Delta_{i}^{h}x\right)}^{2}+{\left(\Delta_{i}^{v}x\right)}^{2}}$$

(14) $$\Phi_{niTV}\left(x\right)=\sum_{i}|\Delta_{i}^{h}x|+|\Delta_{i}^{v}x|$$

where Formulas (13) and (14) represent isotropic and anisotropic regularization, respectively, and $\Delta_{i}^{h}$ and $\Delta_{i}^{v}$ are the horizontal and vertical first-order differential operators. Considering that natural image gradients are typically anisotropic and non-uniform show in Figure 5, we opt for Formula (14). The optimization objective function can be rewritten as:

(15) $$f\left(x\right)=\arg\min\left(\frac{1}{2}||y-{Kx||}^{2}+\Phi_{niTV}\left(x\right)\right)$$

Combining Equations (11) and (15), our model can be expressed by the following formula:

(16) $$f\left(x\right)=\arg\min\left(\frac{1}{2}||y-F^{*}\Sigma Fx{\left|\right|}^{2}+\Phi_{niTV}\left(x\right)\right)$$

This formulation underscores our approach to mitigating the challenges posed by diffractive lenses, leveraging anisotropic TV regularization to counteract the color bias and blurring while preserving essential image features. By optimizing this function, we aim to achieve a balance between fidelity to the observed data and the enforcement of a priori knowledge about natural image characteristics. The result is a reconstructed image that not only closely matches the observed data but also retains the natural appearance and sharpness, despite the inherent limitations of the imaging system. This methodological framework not only exemplifies the application of linear inverse problem-solving to image recovery but also highlights the adaptability of regularization techniques to specific imaging challenges, paving the way for advancements in computational imaging and beyond. We use the TwIST algorithm [31] to optimize the objective function. Proposed by José M. et al., this algorithm improves upon IST (iterative shrinkage/thresholding), enhancing convergence rates. The algorithm employs mean square error (MSE) as an evaluation metric for recovered images, defined as:

(17) $$MSE=\frac{1}{MN}\sum_{i=1}^{M}\sum_{j=1}^{N}{\left[I\left(x,y\right)-\hat{I}\left(x,y\right)\right]}^{2}$$

Additionally, we introduce the correlation coefficient (CC) metric. For two images *A* and *B*, the correlation coefficient is defined as:

(18) $$CC=\frac{\sum_{i}^{N}\left(A_{i}-\bar{A}\right)\left(B_{i}-\bar{B}\right)}{\sqrt{\sum_{i}^{N}{\left(A_{i}-\bar{A}\right)}^{2}\sum_{i}^{N}{\left(B_{i}-\bar{B}\right)}^{2}}}$$

where $\bar{A}$ and $\bar{B}$ are the mean values of the images. For RGB images, we compute this metric separately for each channel.

## 6. Result

**Figure 6:**
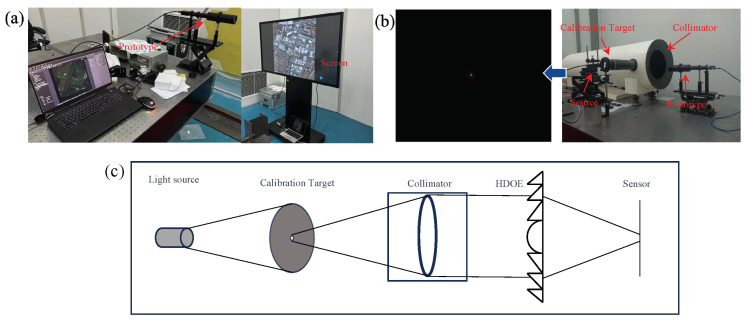
We conducted both indoor and outdoor experiments. (**a**) Illustration of our indoor experimental system, where photos are displayed on a screen and captured using our assembled system. (**b**) System diagram for acquiring the system’s point spread function (PSF) using a parallel light tube. (**c**) Schematic diagram corresponding to (**b**).

**Figure 7:**
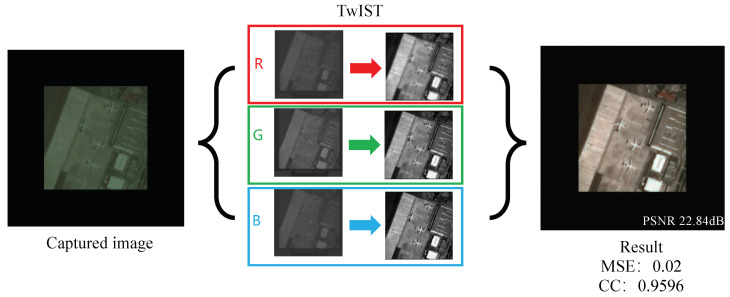
Image reconstruction results. It is worth noting that due to the issue of pixel value matching when photographing the screen, some information will be lost, leading to a decline in the indicators.

In the experimental setup detailed in our study, as depicted in Figure 6a, we utilized a high-resolution monitor as a means to display photographs for the purpose of image acquisition. The images captured by our imaging system, however, did not fully occupy the available frame due to the limitations inherent in aligning the digital display with the camera’s field of view. The point spread function (PSF), a critical component for understanding the system’s imaging capabilities, was collected using an advanced MV-CH250-90UM/C camera. This camera boasts a high resolution of 5120 × 5120 pixels, allowing for detailed capture of the PSF through a parallel light tube, a method which ensures the accuracy and consistency of the PSF data collected. Owing to the practical challenges faced in perfectly aligning the screen with the camera, the resultant captured image dimensions were constrained to 605 × 582 pixels. This limitation necessitated a strategic approach to process and utilize the PSF information effectively while managing the computational demands of the reconstruction process. To this end, we opted to crop the PSF image to a resolution of 2048 × 2048 pixels. This resolution was judiciously chosen to be significantly larger than that of the captured images, thus preserving the essential information of the PSF while facilitating a manageable computational workload. To further refine our image reconstruction process, we employed the technique of cyclic convolution. This involved padding the captured images to match the size of the PSF, thereby enabling us to perform cyclic convolution between the image and the PSF. The iterative reconstruction algorithm employed in our study was the TwIST (two-step iterative shrinkage/thresholding) algorithm. By iterating this algorithm 10 times, we aimed to strike a balance between achieving a high-quality reconstruction and maintaining computational efficiency. The ultimate recovery effect, as illustrated in Figure 7, showcases the efficacy of our methodological choices. The use of cyclic convolution, in conjunction with the TwIST algorithm, facilitated the recovery of images with remarkable clarity and detail, demonstrating the potential of our approach in overcoming the challenges posed by chromatic aberration in diffractive lenses through innovative experimental and computational strategies.

**Figure 8:**
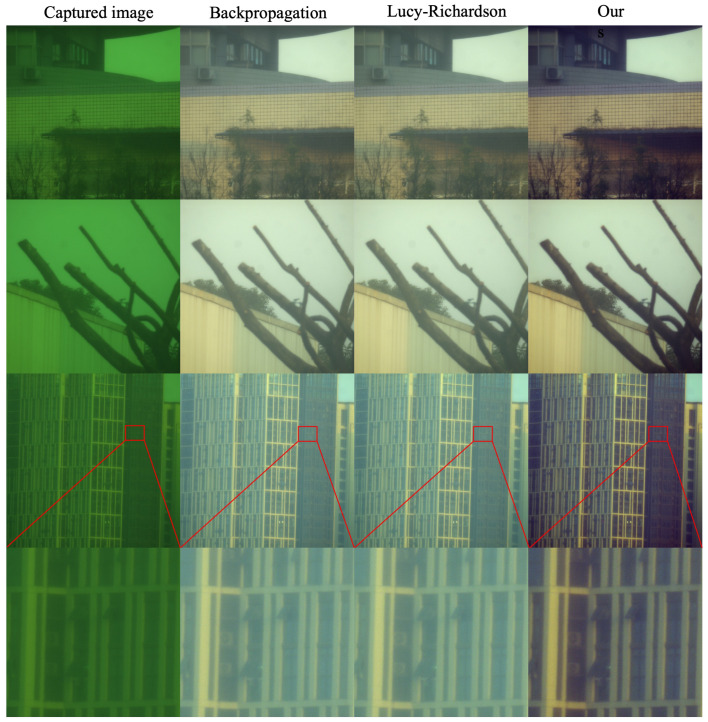
The captured images were processed using our proposed algorithm for chromatic aberration correction and were compared with other algorithms. The results from other algorithms still exhibit a fogging effect, while ours visibly demonstrate superior performance.

**Figure 9:**
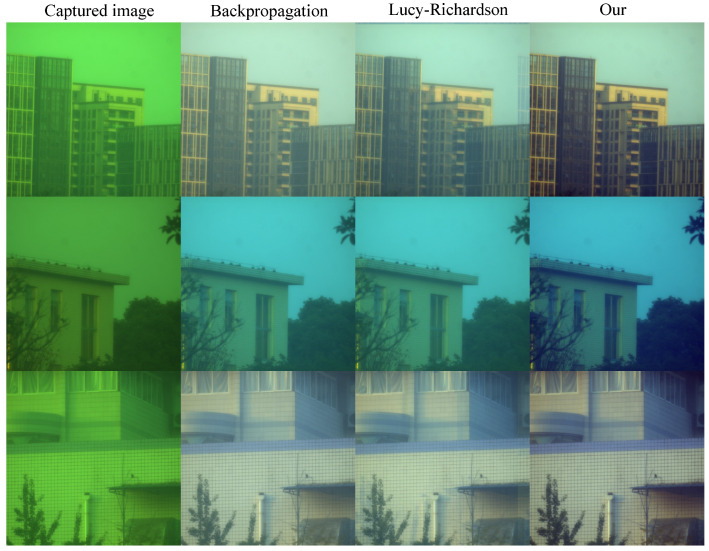
Additional experimental renderings prove the robustness of our algorithm and its advantages over the other two algorithms.

Furthermore, we conducted outdoor scene experiments, using the setup shown in Figure 3c, and compared the results with other PSF-based recovery algorithms, as shown in Figure 8. Our algorithm demonstrates several key advantages in the realm of image reconstruction, particularly when compared with traditional methods such as backpropagation and Lucy–Richardson algorithms. First, the efficiency and accuracy in handling diffraction-limited systems are notable. The algorithm can achieve higher resolution and clarity in the reconstructed images, which is critical for applications requiring fine detail and precision. Another advantage lies in the algorithm’s ability to manage noise effectively. In many imaging scenarios, especially in low-light conditions or when dealing with highly scattering media, noise can significantly degrade the quality of the reconstructed image. Our algorithm incorporates advanced noise reduction techniques that maintain the fidelity of the original signal while minimizing the impact of noise, thus ensuring cleaner, more accurate reconstructions. Furthermore, the algorithm’s robustness against aberrations is a significant benefit. Both the backpropagation and Lucy–Richardson algorithms exhibit a noticeable fogging effect, while our algorithm does not. We also added additional experiments to demonstrate this, as shown in Figure 9.

## 7. Discussion

In this pioneering study, we embarked on the ambitious project of designing and developing a harmonic diffractive lens. This lens was meticulously engineered to operate efficiently across a broad spectrum of wavelengths, specifically from 500 to 800 nm. The foundation of our endeavor was the strategic application of the gradient descent method. This systematic approach was instrumental in optimizing the diffractive structures of the lens to ensure peak performance within the targeted wavelength range. At the core of our investigation was an in-depth analysis of the lens’s focusing capabilities, particularly its ability to handle chromatic aberration—a prevalent obstacle in the realm of diffractive lens systems. Chromatic aberration, a phenomenon characterized by the differential focusing of light wavelengths leading to blurred or distorted images, was a critical focus of our work. When focusing on the target wavelength in the image plane (corresponding to the green channel in this work), other wavelengths (red and blue channels) exhibit a defocusing effect. However, since photon information can still be received, we consider this a unique form of “encoding”. We approached this challenge by conceptualizing chromatic aberration as a unique form of defocusing, occurring independently across individual color channels. In response, we developed a groundbreaking image processing algorithm based on compressed sensing theory. This algorithm was meticulously crafted to reconstruct images free from chromatic aberrations by efficiently merging all-focus images, which were reconstructed from the sparse and incomplete information characteristic of separate channels. This innovation effectively surmounted the inherent limitations imposed by chromatic dispersion in diffractive lenses. A cornerstone of our methodological framework was the strategic employment of the system’s PSF as the measurement matrix within the compressed sensing paradigm. By leveraging the PSF’s intrinsic incoherence, we achieved a comprehensive recovery of information from channels affected by chromatic aberration, thereby not only correcting chromatic aberration but also significantly enhancing imaging quality. The empirical validation of our algorithm through a series of meticulous fabrication experiments underscored the practical viability of our approach, illustrating its superiority over previous computational imaging designs. Our research is further distinguished by the employment of a larger aperture (40 mm) diffractive lens, surpassing the limitations of contemporary end-to-end design frameworks constrained by extensive datasets and considerable computational demands. Hence, our approach broadens the scope of diffractive lens design by sidestepping these constraints.

While our contributions signify a substantial leap forward in correcting chromatic aberration and advancing diffractive lens design, we recognize the ongoing need for refinement. Enhancing image clarity remains a pivotal aim for our future research endeavors. By persistently refining our algorithm and design strategy, we aim to unveil further potential for high-fidelity, chromatic aberration-free imaging, thereby catalyzing new applications and technological advancements in the optical domain. This integration of lens design optimization, advanced image processing algorithms, and innovative fabrication techniques embodies a comprehensive strategy towards achieving high-fidelity, chromatic aberration-free imaging. Such collaborative efforts, as echoed in the work of [32,33], not only reinforce our findings but also pave the way for future innovations in optical imaging technologies. As underscored by [34], the continued exploration of diffractive optics and computational algorithms holds great promise for the field, heralding a new era of optical solutions that enrich both the scientific community and technological applications. Our future work will not be limited to imaging at three wavelengths but will expand to multi-wavelength, that is, multispectral imaging, to capture information across a broader range of frequencies. Through this approach, we aim to achieve a more comprehensive and detailed analysis of target scenes. Multispectral imaging technology can provide richer information than traditional single-wavelength or three-wavelength imaging, including but not limited to the chemical composition of materials, surface textures, and the ability to differentiate between different objects. The development of this technology will significantly enhance our depth and breadth of understanding of complex scenes, thereby playing a crucial role in various fields such as environmental monitoring, medical diagnosis, and the authentication of artworks. Furthermore, we plan to develop more advanced image processing algorithms to handle the multispectral data, addressing the additional complexity introduced by the increase in the number of wavelengths. Our goal is to optimize image quality and accuracy through these algorithms while maintaining efficient computational efficiency to ensure real-time processing and analysis of this data. In summary, we believe that by extending to multispectral imaging and combining it with advanced image processing techniques, we can break through current limitations and begin a new chapter in our understanding of the material world.

## Data Availability

The data that support the findings of this study are available from the corresponding author, Bin Fan, upon reasonable request.
